# Antimicrobial and biodegradable PVA/rutin/CuO nanocomposite film for garlic preservation

**DOI:** 10.1038/s41598-025-18888-w

**Published:** 2025-09-29

**Authors:** Gayathri Gangadharan, Yashoda Malgar Puttaiahgowda, Sonali Gupta, Ananda Kulal

**Affiliations:** 1 Department of Chemistry, Manipal Institute of Technology, Manipal Academy of Higher Education, Manipal, 576104 Karnataka India; 2 Biological Sciences Division, Poornaprajna Institute of Scientific Research, Devanahalli, Bengaluru, 562164 Karnataka India

**Keywords:** PVA, Rutin, CuO nanoparticle, Food packaging, Food preservation, Chemistry, Nanoscience and technology

## Abstract

The growing demand for sustainable and active packaging materials has led to the exploration of biodegradable polymers reinforced with functional nanomaterials. In this study, polyvinyl alcohol (PVA)-based films were synthesized via solution casting method, incorporating copper oxide nanoparticles (CuO NPs) and rutin to improve their physicochemical and antimicrobial properties for food packaging applications. The incorporation of CuO NPs contributed to notable improvements in mechanical strength, water solubility, water vapor permeability, moisture adsorption, and antimicrobial efficacy against *S. aureus*,* E. coli*, and *C. albicans*. PRC-3 film exhibited zone of inhibition of 38.00 ± 0.66, 29.66 ± 0.44, and 26.66 ± 0.44 mm against *S. aureus*,* E. coli*, and *C. albicans*, respectively. The films also showed excellent biodegradability, reinforcing their promise as an environmentally responsible packaging alternative. Rutin release study revealed minimal diffusion into aqueous media, supporting the films potential for prolonged active function. Notably, the practical application test involving garlic storage demonstrated effective preservation of quality for up to 23 days. Altogether, these results underline the potential of PVA/Rutin/CuO (PRC) nanocomposite films as multifunctional, eco-friendly candidates for next-generation active food packaging systems.

**Supplementary Information:**

The online version contains supplementary material available at 10.1038/s41598-025-18888-w.

## Introduction

In the era of globalization, the rapid pace of modern life has significantly altered human lifestyles and dietary patterns, leading to a marked increase in the consumption of packaged foods over traditionally prepared meals. The global food industry is very concerned about the safety and longevity of packaged food^1–4^. Oxidation, microbial spoilage, and metabolic processes are major contributors to the deterioration of various food products. These factors are closely linked to the decline in food quality and safety, which not only influence consumer purchasing behavior and health but also have significant economic implications for the food industry^5,6^. Of particular concern is microbial contamination, which has emerged as a critical threat to food safety^7,8^. The microbial infection caused by packaged food deteriorates the well-being of society both economically and health-wise. The tiny microorganisms are life-threatening in many cases. The capacity of microorganisms to infect people and cause illness varies. Some are benign; some can cause an illness with few symptoms; some may spread across the population and result in a major epidemic sickness, while others may cause significant diseases^9,10^. This is a major issue, and this can be mitigated by the development of antimicrobial food-packaging films. Antimicrobial active packaging delays the initial growth phase of microorganisms. As a result, it helps preserve food quality and safety while extending its shelf life^11^.

Similarly, in the synthesis of packaging films, increasing emphasis is being placed on developing biodegradable polymers, primarily due to the rising concern over plastic waste accumulation and its environmental impact^12^. Biodegradable polymers obtained from renewable resources offer environmentally friendly and sustainable alternatives to conventional commercial plastics^13^. Among them, Polyvinyl alcohol (PVA) is a versatile polymer exhibiting a wide spectrum of properties. It is non-toxic, biodegradable, less expensive, and biocompatible. The outstanding film-forming ability of PVA makes it a valuable material in the food packaging industry^14–16^. Due to these properties, PVA finds its unique position in the food packaging industry. Rutin (3,3’,4’,5,7-pentahydroxyflavone-3-rhamnoglucoside), also known as sophorin or vitamin P, is a citrus flavonoid glycoside. It is commonly found in passionflower, tea, apple, and buckwheat^17^. Rutin exhibits properties like antioxidant, anticarcinogenic, cardioprotective, anti-inflammatory, and many others, which benefit health^18,19^. Rutin is water-insoluble but dissolves in solvents like ethanol^20^. Rutin is a promising functional additive for film formulations, offering antioxidant protection for food products and potential health benefits as a dietary supplement^21^.

In recent days, nanoparticles with inherent antimicrobial properties have been widely used in synthesizing films for food packaging. This incorporation helps to prevent microbial growth, ensuring the quality and extending the shelf life of food products^22^. Also, the high surface area-to-volume ratio of nanoparticles allows them to function as nanofillers within the polymer matrix. This helps in improving the mechanical, barrier, and thermal properties of the films^23^. Copper-based metal oxide nanoparticles (CuO NPs) have attracted significant attention owing to the remarkable physicochemical properties of copper, particularly its capacity to exist in various oxidation states such as Cu^0^, Cu^2+^, and Cu^3+^, represented by forms like elemental copper (Cu), cuprous oxide (Cu_2_O), and cupric oxide (CuO)^24^.

Although numerous studies have investigated the incorporation of CuO NPs and plant-derived compounds like rutin into biodegradable food packaging, most have focused on their individual effects or conventional combinations. In contrast, the present study explores a novel composite system based on polyvinyl alcohol (PVA), copper oxide nanoparticles (CuO NPs), and rutin.Indeed, for the first time, this formulation has been carried out. CuO NPs, which possess stronger antimicrobial efficacy at lower concentrations, and their combination with rutin is hypothesized to create a synergistic effect, enhancing the physicochemical and bioactive properties of the film. Moreover, the current work includes a real-food application (garlic preservation) and assessing biodegradability and controlled release of rutin, offering a more practical and comprehensive understanding of the film’s potential for active food packaging. This research supports the United Nations Sustainable Development Goals, particularly Goal 12 (Responsible Consumption and Production) and Goal 13 (Climate Action), by promoting the development of biodegradable and antimicrobial packaging materials that reduce plastic waste and enhance food preservation, thereby contributing to more sustainable food systems and environmental protection.

## Description of the work

### Materials

PVA was acquired from Loba Chemie Pvt. Ltd. and utilized in its obtained condition. Rutin was procured from BLD Pharm. Copper oxide (CuO) nanoparticles were obtained from Adnano Technologies. Absolute ethanol was obtained from Analytical reagent and used without any purification.

### Film fabrication

A 3% PVA solution was prepared by dissolving 1.5 g of PVA in 50 mL of distilled water under continuous stirring at 80 °C for 45 min. 0.25 g of rutin was dissolved in a 50 mL of ethanol-water mixture (2:1 ratio). PVA and rutin solution were taken in equal proportions and stirred for 3 h to prepare the PVA/rutin (PR) solution. Similar solutions were prepared, and around 1%, 2%, and 3% CuO nanoparticles (Table 1) were incorporated into these solutions and sonicated for 30 min for preparing PRC-1, PRC-2, and PRC-3 films, respectively. Then, it was transferred into Petri plates and kept for drying at 40 °C to achieve films.

In the preparation of the films, 0.25 g of rutin (0.5%) was incorporated. Preliminary trials indicated that 0.5% was the optimal concentration, as lower concentrations reduced the active compound loading, while higher concentrations adversely affected film uniformity. This choice is further supported by a previous study^20^, where rutin was incorporated at 0.2%, 0.4%, and 0.6%. Accordingly, 0.5% of rutin was selected for subsequent experiments.

**Table 1:** Composition of synthesized films.

Sample code	PVA (g)	Rutin (g)	CuO (wt%)
**PVA**	1.5	-	-
**PR**	1.5	0.25	-
**PRC-1**	1.5	0.25	1
**PRC-2**	1.5	0.25	2
**PRC-3**	1.5	0.25	3

### Film characterization

The FTIR spectra of samples were recorded using a Shimadzu-8400 S FTIR spectrophotometer within the range of 400–4000 cm^-1^. The structural properties of CuO NPs and PR-based films were analyzed using a Rigaku Ultima IV X-ray diffractometer with a Cu-Kα radiation source of 1.54 A° wavelength. The diffractogram was obtained within a 2Ɵ range of 10° to 70° at a scan rate of 1°/min under room temperature conditions (27 ± 2 °C). Field Emission Scanning Electron Microscopy (GEMINI 300, Carl Zeiss, Germany) was employed for examining the surface morphology of films.

### Physicochemical properties of films

#### Thickness and tensile strength measurements

Film thickness was measured using a digital micrometer (Mitutoyo, Japan) with a precision of 0.001 mm. The mechanical properties of the films were examined using a Shimadzu EZ-SX Universal Testing Machine according to ASTM-D638-22 standard.

#### Thermogravimetric analysis

Thermogravimetric analysis was carried out using a STA 200 Thermal Analysis System (Hitachi, Japan) to evaluate the thermal stability of the films. Samples were heated from 40 °C to 600 °C at a 10 °C/min heating rate under a nitrogen atmosphere.

#### Moisture adsorption capacity (MAC) and water solubility (WS)

For moisture adsorption analysis, a small modification from Roy et al. method was used^25^. To record the initial dry mass (M_1_) of the film, 2 × 2 cm^2^ samples were dried at 70 °C for 24 h. Then, the films were conditioned under atmospheric conditions for 24 h, and their final mass (M₂) was recorded. The average moisture adsorption was then calculated using the following equation (1):

1 $$\:Moisture\:adsorption\:\left(\%\right)\:=\:\frac{\:M}{{M}_{1}}\times\:\:100$$

Where M is M_2_-M_1_.

For analysing the water solubility, films of 2 × 2 cm^2^ were dried at 70 °C overnight, and their initial mass (M_1_) was noted^26^. For 24 h, the films were submerged in 20 mL of distilled water, and then excess water was drained. The films were dried at 70 °C, and the final mass(M_2_) was noted. The average water solubility was calculated using equation (2):

2 $$\:Water\:solubility\:\left(\%\right)\:=\:\frac{\:{M}_{1}-{M}_{2\:}}{{M}_{1}}\times\:\:100$$

#### Water vapor permeability (WVP)

A modified version of the method described by Gasti et al.^22^ was employed to evaluate the water vapor permeability (WVP) of the prepared films. Glass bottles with an average mouth diameter of 1.2 cm and a depth of 8.0 cm were used for the test. Each bottle was filled with anhydrous calcium chloride (CaCl_2_) to maintain a relative humidity (RH) of 0%, leaving a 1 cm gap between the desiccant and the film sample. The mouth of each bottle was sealed with the film specimen and tightly secured using Teflon tape to prevent vapor leakage. The sealed bottles were then placed in a desiccator containing distilled water to maintain a 100% RH environment. The weight gain of each bottle, resulting from water vapor permeation through the film, was recorded at 24 h intervals for four consecutive days. Linear regression analysis (with R^2^ ≥ 0.982) was applied to the time vs. weight gain data to determine the slope. The water vapor transmission rate (WVTR) was calculated by dividing the slope (g/h) by the area of the bottle mouth (m^2^). The water vapor permeability (WVP) was subsequently computed using equation (3):

3 $$\:WVP=\:\left(\left(\frac{WVTR}{P}\right)\left(RH1-RH2\right)\right)\times\:\chi\:$$

Where P is the vapor pressure of water at 25 °C, RH1 is the relative humidity inside the test bottle, RH2 is the relative humidity outside the test bottle and χ is the thickness of the film (m).

#### Soil burial test (SBT)

Film samples measuring 2 × 2 cm^2^ were dried at 40 °C, and their initial weight (W_i_) was recorded. The films were buried in soil at a depth of 5 cm. To ensure a moist environment, water was sprayed onto the soil every 72 h. After 15 days, the films were recovered, rinsed with distilled water, and for 24 h, the films were dried in a hot air oven. The final weight of the samples (W_f_) was then recorded, and the percentage of degradation was calculated using the following equation (4):

4 $$\:Degradation\:rate\:\left(\%\:\right)\:=\:\frac{\:W}{{W}_{i}}\times\:100$$

Where W is W_i_-W_f_.

#### Release study of rutin

The release of rutin from the film (PRC-2) in water was evaluated following the method described by Roy and Rhim (2021)^27^, with slight modifications. A film sample measuring 2.5 cm × 2.5 cm was immersed in 20 mL of distilled water in a 100 mL conical flask and maintained at 25 °C under gentle shaking. At predetermined time intervals (0, 60, 120, 180, and 240 min), 1 mL of the release medium was withdrawn, and its absorbance was measured at 360 nm using a UV–Visible spectrophotometer. To quantify the release, a calibration curve of rutin in the concentration range of 5–30 µg/mL was plotted. The absorbance values obtained from the release medium were converted to concentrations using this calibration curve, and the total rutin released was calculated. The release results were then expressed as the amount of rutin released per unit film area (µg/mm^2^), consistent with the standard representation in the literature.

#### Antimicrobial activity

##### Microbial cell culture

Nutrient agar and nutrient broth served as the growth media. The bacterial strains *E. coli* (MTCC 1687), *S. aureus* (MTCC 3160), and the fungal strain *C. albicans* (MTCC 7523) were inoculated and incubated in nutrient broth at 37 °C for 12 h. The microbial culture was then prepared by diluting 100 µL of the 0.5 McFarland standard culture in 10 mL of sterile Milli-Q water.

##### Disc diffusion method

Disc diffusion method was employed to evaluate the antimicrobial activity against *E. coli*,* S. aureus*, and *C. albicans*. Autoclaved nutrient agar medium was poured into petri plates and allowed to solidify in a laminar air flow chamber aseptically. For each agar plate, 100 µL of microbial culture was carefully added and evenly spread using an L-shaped sterile glass rod. The discs of PR, PRC-1, PRC-2, and PRC-3 films (~ 6 mm size) were made using the punching machine, and two discs of each sample were kept on the microbial lawn and incubated at 37 °C for 6 h. One disc of each antibacterial (Cephalothin, 30 mcg) and antifungal (Fluconazole, 10 mcg) standards were used along with the test samples. After the incubation, the zone of inhibition was measured using a student scale^28–30^.

##### Anti-microbial activity by immersion and spread plate method

The antimicrobial activity of the polymer nanocomposite was evaluated against various bacterial and fungal strains by immersing samples in microbial culture and testing for microbial counts using the spread plate method. 900 µL of nutrient broth is taken in a 2 mL Eppendorf tube, 100 µL of diluted microbial suspension was added to each tube, later one sample disc (~ 6 mm size) of polymer sample (PR, PRC-1, PRC-2, and PRC-3) were added to the tubes containing media and incubated at 37 °C for up to 12 h with continuous shaking. One control tube containing the same media without the polymer was used as a positive control. One disc of each antibacterial (Cephalothin, 30 mcg) and antifungal standards (Fluconozole,10 mcg disc) were used as standards.

Following 12 h of incubation, 10 µL of the media from each tube was diluted with 990 µL of sterile Milli-Q water (10^2^-fold dilution). Then, 100 µL of this diluted sample was spread onto nutrient agar plates using a sterile L-shaped glass rod. The plates were incubated at 37 °C for 4–12 h to observe colony formation. Colony-forming units (CFUs) were counted in all samples, standard, and control plates to calculate the percent reduction in colonies in the samples and standard plates compared to the control. This data was then used to assess the polymer’s antimicrobial activity.

### Packaging study of garlic clove

In this study, garlic was utilized to assess the packaging and shelf-life performance of the developed active packaging films. Fresh garlic cloves were procured with care from a local marketplace in Manipal, Karnataka, ensuring quality and authenticity. Garlic cloves were wrapped in the synthesized films PR, PRC-1, PRC-2, and PRC-3, while a control sample was left unwrapped. All samples were stored at room temperature. The garlic’s texture and appearance were regularly observed over time to assess the impact on its shelf life.

### Statistical analysis

Measurements, performed in triplicate (*n* = 3), were subjected to statistical analysis using Origin Pro 2025 software. Data were evaluated by one-way ANOVA followed by Tukey’s post hoc test, with statistical significance set at *p* ≤ 0.05. Results are reported as mean ± standard deviation (SD).

## Results and discussion

### FT-IR analysis

**Fig. 1:**
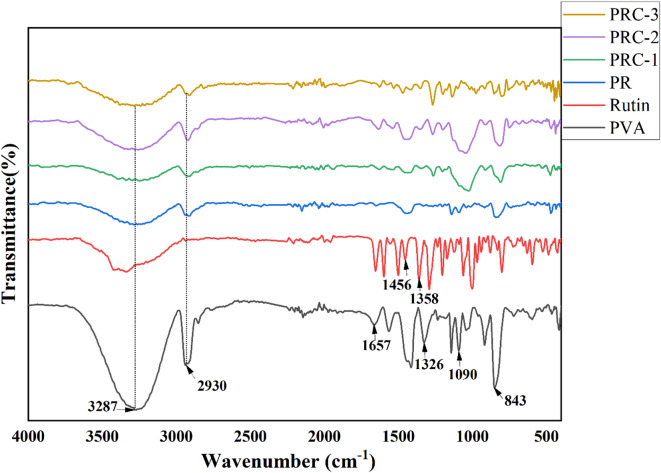
FTIR spectra of pure PVA, rutin, PR, PRC active films.

The FTIR spectrum of pure PVA film, as shown in Fig. 1, displays characteristic absorption bands corresponding to its functional groups. A broad and intense absorption peak observed at 3287 cm^−1^ is attributed to the stretching vibrations of hydroxyl (-OH) groups, which arise due to inter and intramolecular hydrogen bonding within the polymer matrix^31^. The absorption peak at 2930 cm^−1^ is indicative of the asymmetric stretching vibration of methylene (-CH_2_) groups present in the PVA backbone. A band at 1657 cm^−1^ corresponds to the bending vibration of the -OH groups, typically associated with absorbed water or hydrogen-bonded hydroxyl groups. The minor peak observed at 1326 cm^−1^ is assigned to the bending vibration of the C-O-H group, while the band at 1090 cm^−1^ is characteristic of C–O stretching vibrations. The peak at 843 cm^−1^ is related to the C–H rocking vibrations of the PVA chains, confirming the semi-crystalline nature of the polymer^32–34^. The FTIR spectrum of pure rutin shows a broad absorption band centered around 3423 cm^−1^, which signifies the presence of phenolic -OH groups and indicates strong hydrogen bonding interactions. The band at 2936 cm^−1^ corresponds to the C-H stretching vibrations of the -CH_2_ groups. The prominent peak at 1456 cm^−1^ is attributed to the stretching vibrations of the carbonyl (C = O) group within the flavonoid structure of rutin. Another characteristic band at 1358 cm⁻¹ is assigned to the C-OH stretching vibrations, further confirming the polyphenolic nature of rutin^20,35^.

**Scheme 1:**
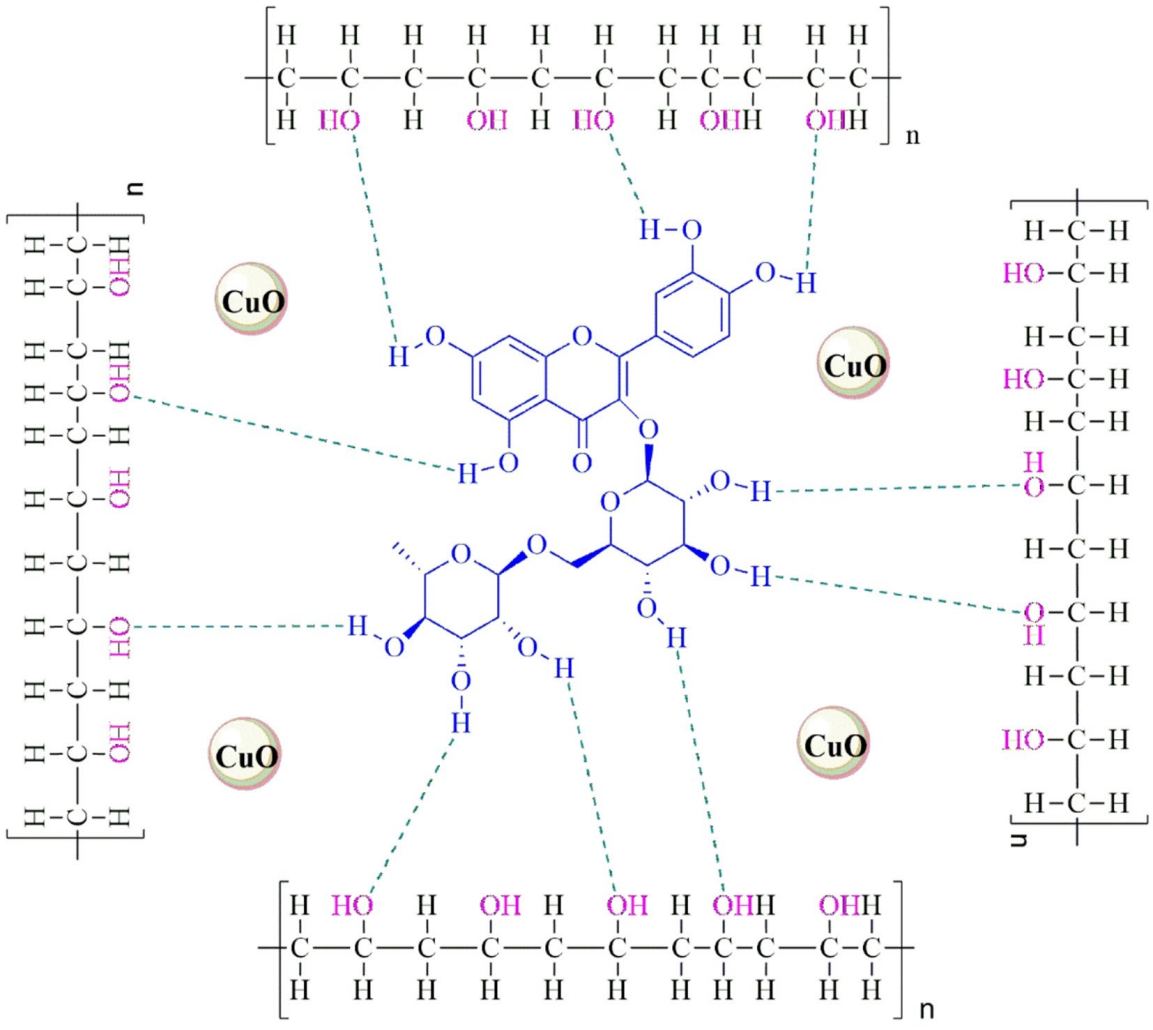
Plausible interaction between PVA, rutin, and CuO NPs.

Upon incorporation of rutin into the PVA matrix, the resulting spectrum (PR film) exhibits a noticeable shift in the –OH stretching band from 3287 cm^−1^ to 3216 cm^−1^. This shift implies the formation of hydrogen bonding interactions between the hydroxyl groups of PVA and the phenolic -OH groups of rutin, suggesting good compatibility and molecular interaction between the two components^20^. Further modification of the PR film with CuO nanoparticles (resulting in PRC-1, PRC-2, and PRC-3) did not introduce any new characteristic peaks, indicating that the addition of CuO did not significantly alter the chemical structure of the PVA/rutin matrix. The major absorption bands observed in PRC films remain similar to those of the PR film, signifying that the incorporation of CuO nanoparticles mainly contributes to physical interactions rather than forming new chemical bonds with the polymer or rutin components (Scheme 1**)**. This observation is consistent with literature reports where metal oxide nanoparticles are dispersed in polymer matrices without altering the primary functional group signatures^36^. Overall, the FTIR analysis confirms the successful incorporation of rutin and CuO into the PVA matrix, with evident molecular interactions between PVA and rutin, while CuO remains chemically inert within the blend^37^.

### XRD

**Fig. 2:**
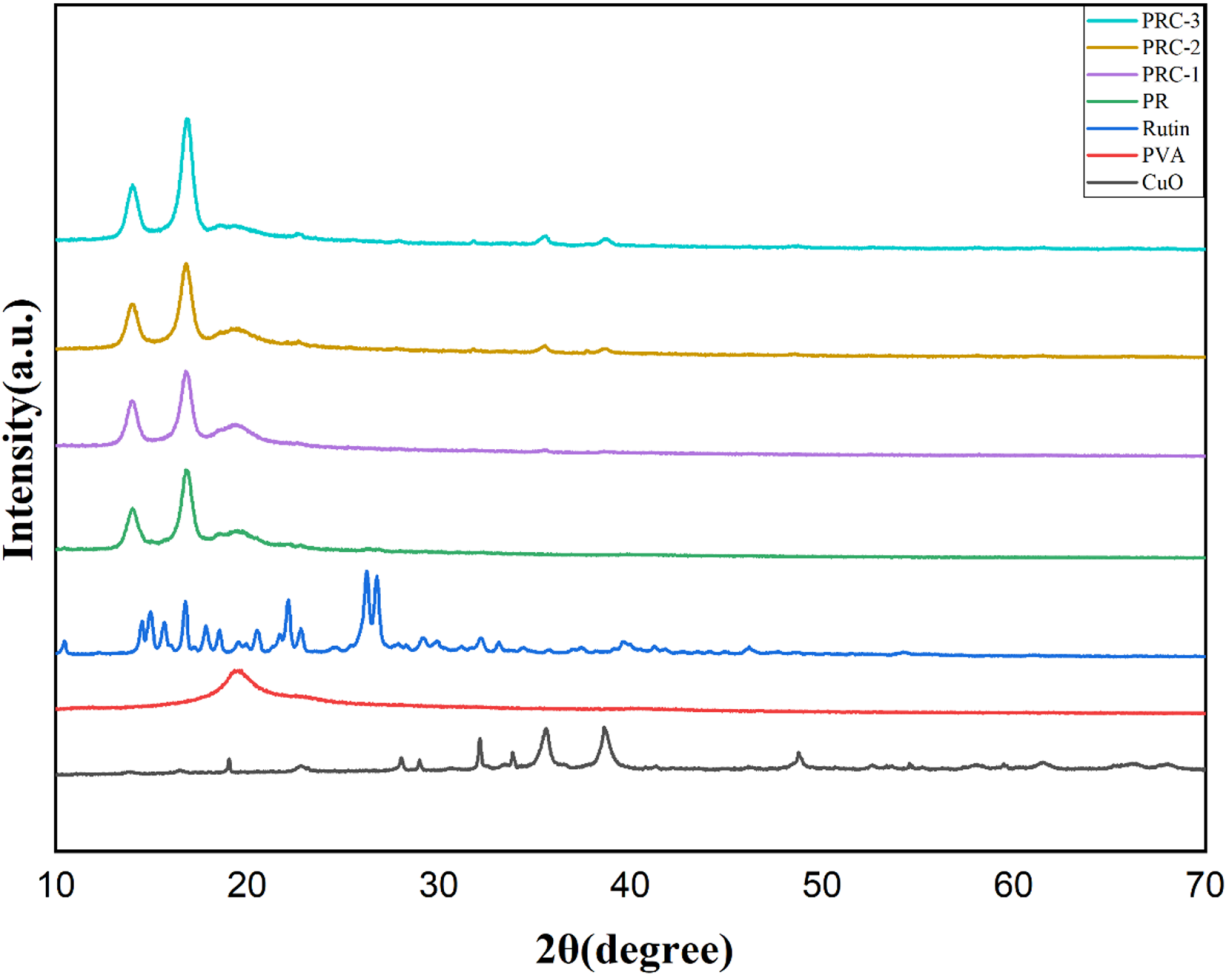
XRD diffraction patterns of CuO, PVA, rutin, PR and PRC films.

The XRD analysis effectively observes the morphology, crystal structure, and chemical composition of active films in packaging material design. Figure 2. displays XRD scans of CuO, PVA, rutin, PVA/rutin blend, and films incorporated with different amounts of CuO in PVA/rutin blend. On examining the diffraction patterns of CuO NPs, it exhibited peaks at 16.46°, 22.9°, 28.08°, 35.50°, 38.64°, and 52.66° corresponding to (020), (021), (110), (002), (111), and (113) planes of CuO^38^. The diffraction peak of pure PVA observed at 2Ɵ=19.6° represents a semicrystalline nature and is a characteristic strong crystalline peak, attributed to the hydrogen bonding between the hydroxyl groups of the PVA chains^39^. Rutin has several crystalline diffraction peaks. The neat PR films exhibited characteristic diffraction peak at 19.6° which corresponds to PVA and peaks at 14.1° and 16.8° indicates rutin. In PRC-1 film due to the lower concentration of CuO NPs, the characteristic peaks of CuO NPs are very insignificant. In PRC-2 and PRC-3, 35.5° and 38.7° indicate CuO NPs in the PVA/rutin matrix^25^.

### Surface morphology

**Fig. 3:**
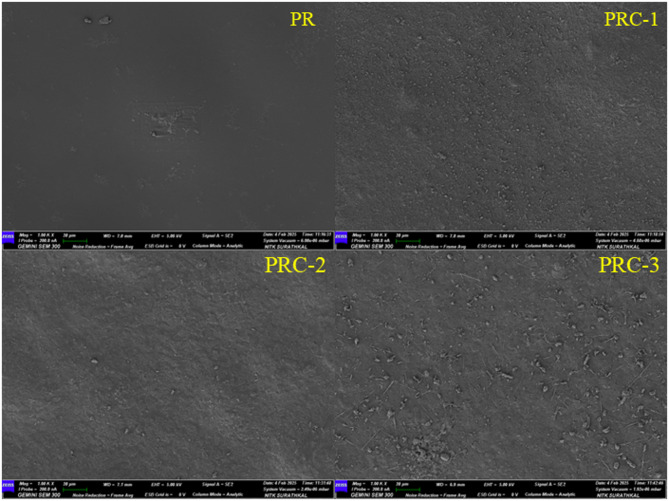
FESEM images of PR and its nanocomposites.

The PR films had smooth surface morphology, and on incorporation of CuO NPs, surface roughness was observed due to the dispersion of the nanoparticles in the PR matrix (Fig. 3.**)**. The agglomeration of CuO NPs was more visible in PRC films, and this agglomeration is more pronounced in PRC-3 films. This agglomeration may be ascribed to the higher concentration of CuO NPs in the PR polymer matrix^40^. An increased concentration of CuO NPs led to the formation of a more compact structure in the resulting bio-nanocomposites^22^.

### Thickness and tensile strength measurements

Film thickness is a critical parameter influencing key attributes such as physical integrity, mechanical strength, and barrier performance. The thickness of nanocomposite films is affected by several factors, including the preparation method, the type of nanoparticles used, and the interactions between biopolymers and nanoparticles^41^. PR film had a thickness of 0.0882 ± 0.01 mm, (Table 2) which is the minimum thickness among all films. The thickness of PRC nanocomposite films increased notably to 0.1238 ± 0.02, 0.1456 ± 0.01, and 0.1906 ± 0.02 mm with the incorporation of 1%, 2%, and 3% CuO NPs, accordingly. The observed increase in film thickness reflects an elevated total solid content resulting from the incorporation of CuO NPs. A similar trend was observed in kodo millet starch/gum tragacanth/CuO films^42^ and chitosan/copovidone/Ag nanocomposite film^36^.

**Fig. 4:**
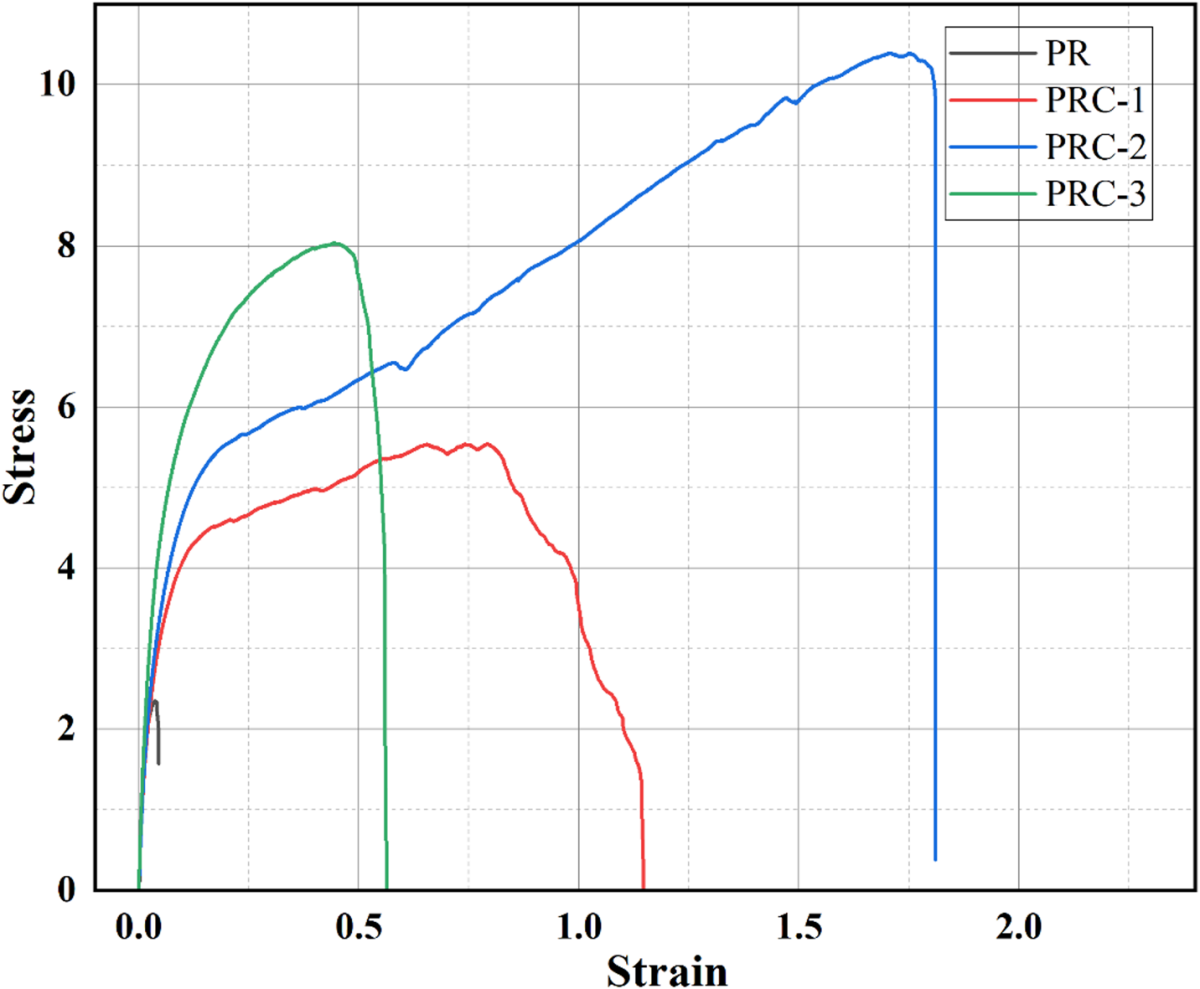
Stress-strain curve of prepared PR and PRC films.

On analyzing the tensile strength, pristine PR film exhibited a tensile strength of 2.71 ± 0.52 MPa. Incorporation of CuO NPs led to a significant increase in tensile strength, reflecting strong interactions between the PR polymer matrix and the nanoparticles, facilitated by their large surface area, as well as reinforcement from intermolecular hydrogen bonding. The PRC-1 film exhibited a tensile strength of 5.32 ± 0.35 MPa, PRC-2 exhibited 10.6 ± 0.29 MPa, and PRC-3 exhibited 7.63 ± 0.65 MPa **(**Fig. 4; Table 2**)**. The differences among the films are statistically significant, with PRC-2 exhibiting the highest tensile strength. The slightly lower tensile strength of PRC-3 than PRC-2 could be due to the presence of voids in the composite, which act as stress concentrators, as well as the aggregation of particles when their concentration is high^22^.

**Table 2:** Thickness, tensile strength, MAC, and WS of PVA/rutin-based composite films. Values are expressed as mean ± standard deviation (n = 3). Within the same column, values sharing the same letter (a–d) are not significantly different (p<0.05).

Sample Name	Thickness (mm)	Tensile strength (MPa)	MAC (%)	WS (%)	WVP×10^–10^ (gm^−1^s^−1^Pa^−1^)	Biodegradability (%)
**PR**	0.0882 ± 0.01	2.71 ± 0.52^d^	10.47 ± 0.10^a^	32.25 ± 0.17^a^	4.58 ± 0.03^a^	41.88 ± 0.55^a^
**PRC-1**	0.1238 ± 0.02	5.32 ± 0.35^c^	10.07 ± 0.08^a^	18.73 ± 0.10^b^	3.39 ± 0.12^b^	35.18 ± 0.32^b^
**PRC-2**	0.1456 ± 0.01	10.6 ± 0.29^a^	9.26 ± 0.11^b^	17.23 ± 0.05^c^	3.07 ± 0.06^c^	32.21 ± 0.52^c^
**PRC-3**	0.1906 ± 0.02	7.63 ± 0.65^b^	8.76 ± 0.26^b^	15.71 ± 0.10^d^	2.88 ± 0.09^c^	27.49 ± 0.42^d^

### Thermogravimetric analysis (TGA)

**Fig. 5:**
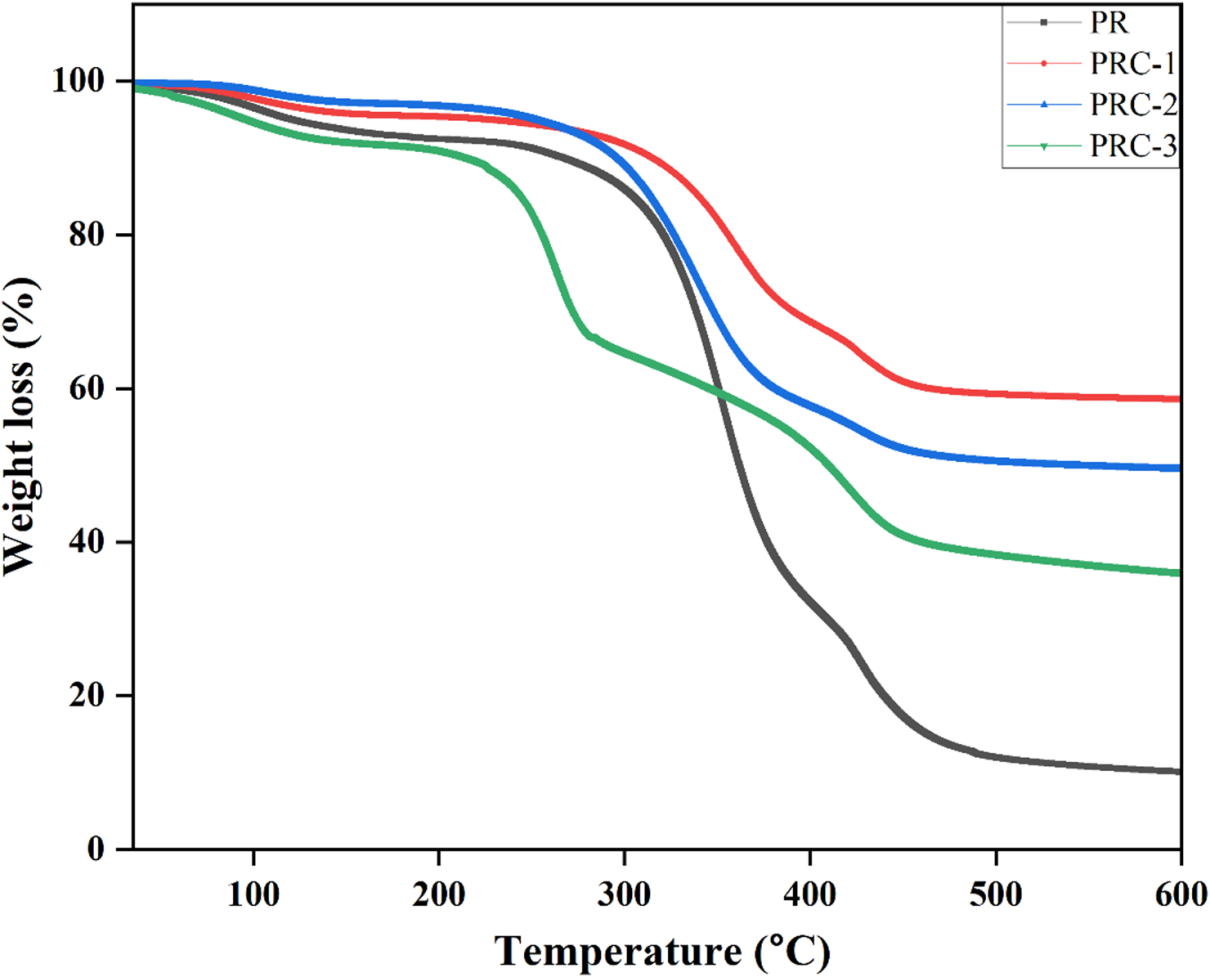
TGA curve of PR, PRC-1, PRC-2 and PRC-3 films.

Thermogravimetric analysis (TGA) was performed to investigate the thermal degradation behavior of the pristine PVA-rutin film (PR) and its nanocomposite variants containing CuO nanoparticles at various concentrations of 1% (PRC-1), 2% (PRC-2), and 3% (PRC-3). The TGA thermograms revealed a characteristic multi-step degradation profile for all samples. The initial weight loss observed at ∼150 °C is attributed to the evaporation of free and bound water **(**Fig. 5.**)**. The second degradation stage, occurring between 200 °C and 350 °C, corresponds to the degradation of the PVA and rutin. Similar thermal behavior has been reported by Narasagoudr et al.^20^, in their study on rutin-incorporated chitosan/PVA composite films for food packaging applications. The final degradation stage, occurring beyond 400 °C, is associated with the breakdown of the PVA main polymer chain. Analysis of the maximum degradation temperatures revealed that the pristine PR film exhibited thermal stability up to 476 °C. Upon incorporation of CuO NPs, a slight reduction in the degradation temperatures was observed for PRC-1, PRC-2, and PRC-3, degrading at 453 °C, 454 °C, and 444 °C, respectively. Despite the decrease in decomposition temperatures, the residual weight till 600 °C was higher for the CuO-reinforced films compared to the pristine PR film, indicating enhanced char formation. This suggests that the incorporation of CuO NPs improved intramolecular interactions within the matrix, contributing to the overall thermal stability of the nanocomposite network. These observations are in agreement with the findings of Suryavanshi et al., who reported similar thermal enhancement in kodo millet starch/gum tragacanth/CuO nanocomposites^42^.

### Moisture adsorption capacity (MAC) and water solubility (WS)

Table 2 shows the water solubility of PR and PRC films. Water solubility is affected by the number of free hydroxyl groups in the polymer matrix, as they aid in hydrogen bonding interactions between the film and water^20^. The PR film had a water solubility of 32.25 ± 0.17%. In the case of PRC-3 film, it demonstrated a water solubility of 15.71 ± 0.10% (Fig. S1). The gradual decrease in water solubility with increasing CuO content indicates that the reduction is statistically significant and highlights the effect of CuO NPs in decreasing the hydrophilicity of the films. This trend can be attributed to strong interactions between CuO NPs and the hydroxyl groups of PVA and rutin. Enhanced hydrogen bonding and electrostatic interactions strengthened the polymer chain network, reducing the availability of hydroxyl groups to interact with water molecules and consequently enhancing the hydrophobicity of the film^25,42^.

Table 2 illustrates the moisture adsorption capacity (MAC) of the synthesized films. The moisture adsorption values of films varied according to the amount of CuO NPs incorporated into the PR matrix. PR film exhibited a high value of MAC. This can be attributed to the increased availability of the free hydroxyl group. Upon incorporation of CuO NPs, the moisture adsorption capacity decreased, with PRC-2 and PRC-3 showing significantly lower values of MAC in comparison to PR^25^. This statistically significant reduction highlights the potential of CuO NPs to interact with the free hydroxyl groups of PVA and rutin, consequently diminishing the interaction between polymers and water molecules in the film matrix^42^ (Fig. S1).

### Water vapor permeability

Water vapor permeability (WVP) is a critical property of packaging materials, as it governs the transfer of moisture from the external environment to the packaged product. Minimizing WVP is essential for maintaining the freshness, texture, and shelf life of food items. A lower WVP value is therefore desirable to effectively limit moisture ingress. Several factors can influence the WVP of a material, including the chemical structure, polarity of the polymer matrix, the degree of polymer chain entanglement, the hydrophobic or hydrophilic nature of the material, and the type and concentration of incorporated fillers or additives^22,43^.

In the present study, the PVA/rutin (PR) film exhibited a WVP of 4.58 ± 0.03 × 10^−10^ gm^−1^s^−1^Pa^−1^ (Table 2). This relatively high value can be attributed to the abundance of hydrophilic -OH groups present in both PVA and rutin, which tend to absorb and facilitate the transmission of water vapor. Upon incorporating CuO NPs, a gradual decrease in WVP was observed. The PRC-1 film exhibited a WVP of 3.39 ± 0.12 × 10^−10^ gm^−1^s^−1^Pa^−1^, while PRC-2 and PRC-3 demonstrated further reductions to 3.07 ± 0.06 × 10^−10^ and 2.88 ± 0.09 × 10^−10^ gm^−1^s^−1^Pa^−1^, respectively. The decreasing trend in WVP values with increasing CuO NP content suggests that the nanoparticles play a significant role in enhancing the film’s moisture barrier properties. The decrease in WVP is statistically meaningful, with the largest reduction observed between PR and PRC-1. For the present study, the PRC-3 film exhibited excellent results. Being inherently hydrophobic, CuO NPs reduce the overall hydrophilicity of the film matrix. Moreover, their presence interferes with the micro-scale pathways through which water vapor typically diffuses, thus forming a denser, more compact film structure. This phenomenon can be attributed to the formation of a complex diffusion pathway that hinders water vapor transmission, due to the uniform dispersion of impermeable CuO NPs within the PVA/rutin matrix. Additionally, the nanoparticles likely establish strong hydrogen bonding interactions with the polymer chains, further reinforcing the matrix and limiting molecular mobility. Notably, the WVP values obtained in this study are lower than those reported by Gunaki et al. for Chitosan/hydroxypropyl cellulose/CuO films^25^, further validating the effectiveness of CuO NPs incorporation in enhancing the barrier performance. In addition, previously reported by Narasagoudr et al.^20^ for PVA/chitosan/rutin composite films have shown higher WVP values than the films developed in the present study, highlighting the effectiveness of our formulation in minimizing moisture permeability. These findings suggest that the incorporation of CuO NPs not only enhances the structural compactness of the PVA/rutin matrix but also significantly improves its water vapor barrier properties. As a result, the developed nanocomposite films hold strong potential for use in food packaging applications, where maintaining product quality and shelf life is essential.

### Soil burial test

To mitigate environmental pollution associated with the post-consumer disposal of plastic food packaging, it is essential that packaging materials are capable of degrading under ambient environmental conditions. Consequently, the biodegradability of the fabricated bio-nanocomposites was assessed through soil burial tests, with degradation examined as a function of weight loss over time^44^. The biodegradability of the PVA/rutin (PR) film was observed to be 41.88 ± 0.55%, indicating a moderate level of degradation under the tested conditions **(**Table 2**)**. However, with the incorporation of CuO NPs, a gradual decrease in biodegradability was noted. The PRC-1, PRC-2, and PRC-3 films exhibited biodegradability values of 35.18 ± 0.32%, 32.21 ± 0.52%, and 27.49 ± 0.42%, respectively (Fig. S1). This trend is statistically significant as indicated by the differing letters (a–d), suggesting that increasing the concentration of CuO NPs negatively affects the film’s biodegradability. This reduction can be ascribed to the enhanced compactness of the polymer matrix, likely due to enhanced hydrogen bonding between the polymer chains and CuO NPs. Moreover, the intrinsic antimicrobial properties of CuO NPs may inhibit microbial activity, further contributing to reduced biodegradation. These findings are in line with previous reports, such as the study by Gunaki et al.^25^, who synthesized chitosan/hydroxypropyl cellulose/CuO films and observed a comparatively lower biodegradation rate than the current PVA/rutin/CuO films. The relatively higher biodegradability of the present films, despite the presence of CuO NPs, suggests a favorable balance between functional enhancement and environmental degradability. This characteristic is especially significant in the context of sustainable packaging solutions, where material performance must be optimized without compromising environmental safety.

### Release study of rutin

**Fig. 6:**
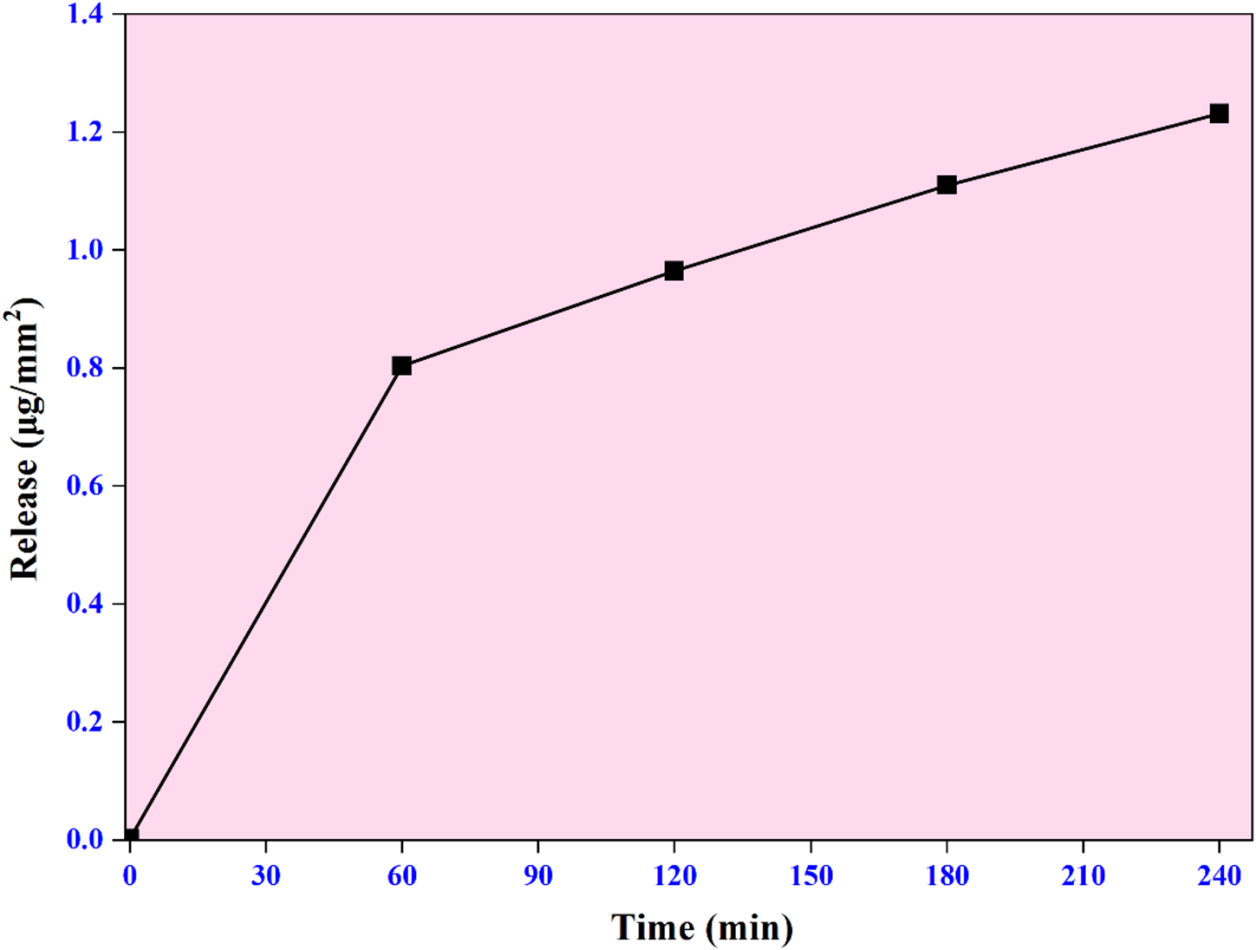
Release study of rutin in water from synthesized PRC-2 film.

The release study of rutin from the PRC-2 film revealed a relatively low diffusion of the compound into the aqueous medium. After 60 min of immersion in water, the amount of rutin released was 0.8040 µg/mm^2^ (Fig. 6.). This gradually increased to 0.9646, 1.1097, and 1.2315 µg/mm^2^ at 120, 180, and 240 min, respectively. The overall release of rutin was significantly lower compared to previously reported films based on gelatin/chitosan matrices incorporated with cinnamon essential oil and rutin^27^. It was also noted that the highest amount of rutin released (1.2315 µg/mm^2^ or 12.315 mg/dm^2^) at 240 min was observed to be within the overall migration limits of < 10 mg/dm^2^ and < 60 mg/dm^2^, indicating its compatibility for food packaging applications^32^. This limited release behavior could be attributed to the strong intermolecular interactions among polyvinyl alcohol (PVA), rutin, and CuO NPs, which may hinder the mobility and diffusion of rutin. Furthermore, the release rate is influenced by multiple factors, including the solubility of the active compound, the degree of swelling and structural integrity of the polymer matrix, and the diffusivity of the bioactive compound within the film network^45^.

### Antimicrobial activity

**Fig. 7:**
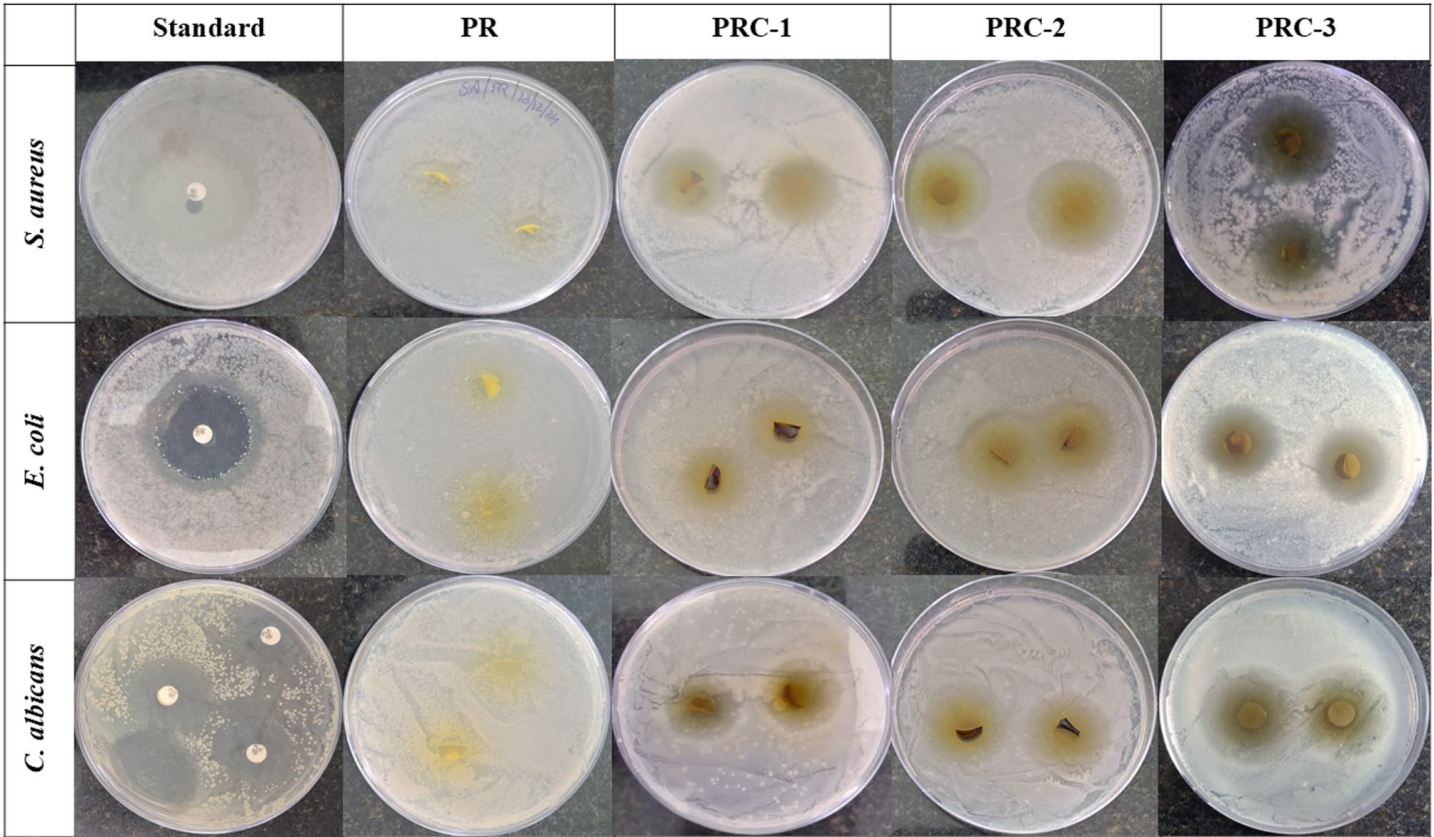
Microbial growth inhibition activity of standard, PR, and PRC films.

 Packaging material infused with antimicrobial agents can improve food protection by preventing microbial contamination and extending their shelf life. Food packaging films with inherent antimicrobial activity are the demand in the present era. Thus, the synthesized PR, PRC-1, PRC-2, and PRC-3 films were evaluated for foodborne pathogens like *E. coli*,* C. albicans*, and *S. aureus*. Figure 7, **Table ****S1****.** indicates the zone of inhibition. The PR films exhibited no antimicrobial activity. On analyzing the zone of inhibition, it was clear that the PRC-3 film had the highest antimicrobial activity. This is due to the presence of higher concentrations of CuO NPs in the polymer matrix. Similarly, by the spread plate method also when colony-forming units were analyzed, a decrease in colony-forming units was observed (**Table S2**,** Fig. S2**).

The oxidation state of CuO is believed to influence its antibacterial properties. As copper in CuO is present in its highest oxidation state (Cu²⁺), it readily releases Cu²⁺ ions from the nanoparticle surface. Inside bacterial cells, these ions are reduced to Cu⁺ by interaction with sulfhydryl groups, leading to oxidative stress through the production of reactive oxygen species (ROS) like superoxide anions and hydroxyl radicals. Additionally, CuO NPs exhibit potent antimicrobial effects by directly interacting with bacterial cell membranes. Due to the presence of nanoscale pores in the membrane, appropriately sized and charged nanoparticles can infiltrate the cells. This intrusion may disrupt vital cellular functions, such as protein and DNA activity, or induce cell death through the production of reactive oxygen species (ROS). Conversely, the amount of copper ions released is minimal and does not reach sufficient levels to exert toxic effects on bacteria, making its impact negligible. Instead, the antibacterial efficacy of CuO is primarily driven by reactive oxygen species and strong protein binding^46^.

### Packaging application

**Fig. 8:**
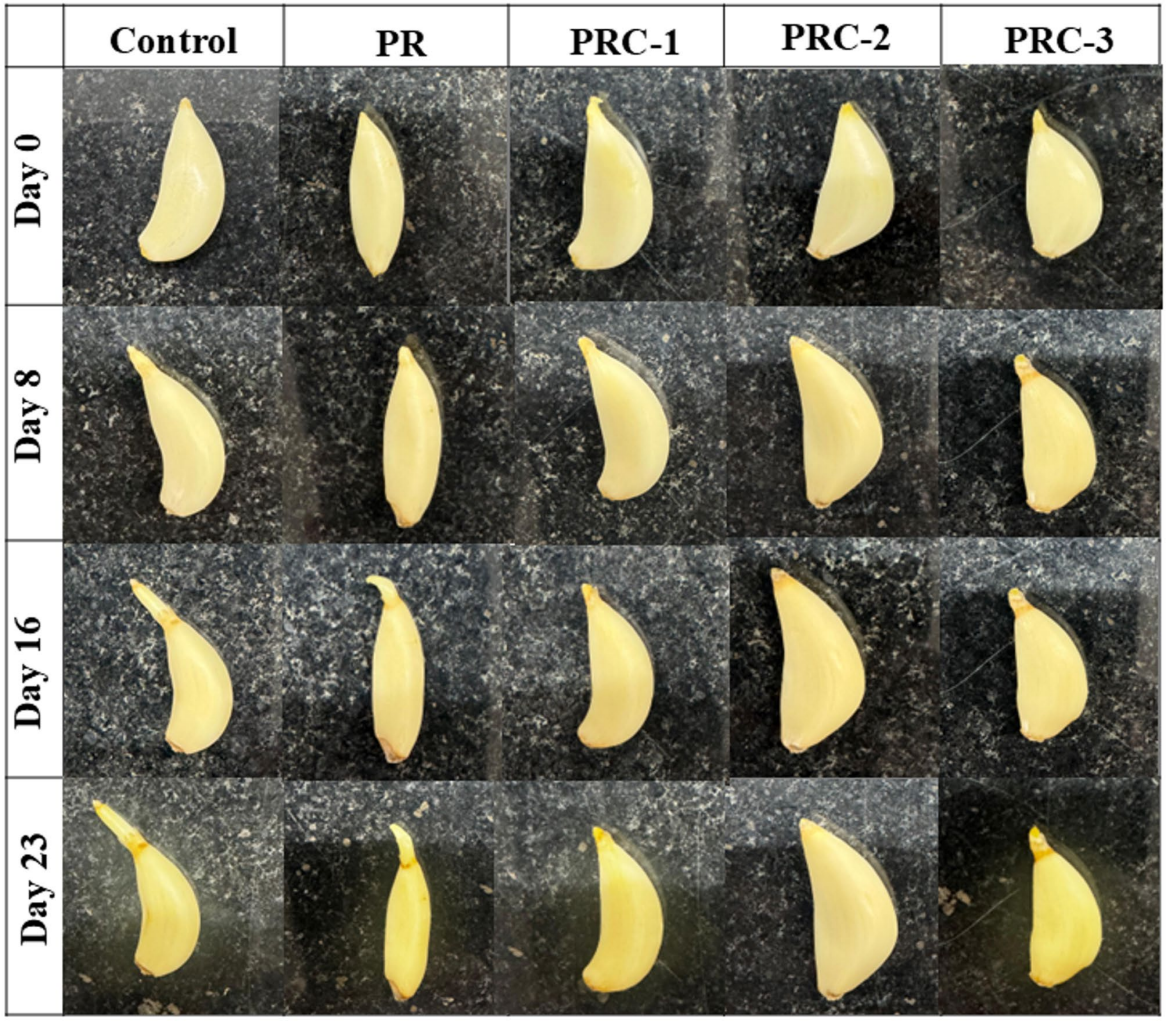
Visual inspection of the appearance of garlic unwrapped, wrapped with PR, PRC-1, PRC-2, and PRC-3 films.

A study was conducted to evaluate the effectiveness of different films for food packaging using garlic cloves as a test sample **(**Fig. 8.**)**. Unwrapped garlic cloves served as the control, while the experimental samples were wrapped in PR, PRC-1, PRC-2, and PRC-3 films. Photographs were taken at regular intervals to monitor visual changes during storage. All samples were placed at room temperature under ambient conditions, and the effectiveness of the films in preserving the quality of garlic was assessed over time. A visible change in color, freshness, and growth of the shoot was more in the film placed without wrapping, and also in the case of garlic wrapped using PR film. The garlic wrapped in PR film was more spoiled comparatively. Garlic cloves packed in PRC-1, PRC-2, and PRC-3 retained quality similar to that observed on day 0, with freshness sustained through day 23. Notably, samples wrapped in PRC-1 and PRC-2 demonstrated superior freshness compared to those in PRC-3, and the most prominent result was observed for PRC-2 film in extending the shelf life of preserved garlic up to 23 days.

## Conclusion

An eco-friendly polymer nanocomposite film based on PVA/rutin/CuO was successfully synthesized and systematically characterized. PRC-3 film exhibited barrier properties, including reduced moisture absorption capacity (MAC) (8.76 ± 0.26%), water vapor permeability (WVP) (2.88 ± 0.09 × 10^−10^gm^−1^s^−1^Pa^−1^), and water solubility (WS) (15.71 ± 0.10%). PRC-2 demonstrated the highest tensile strength (10.6 ± 0.29 MPa). The films also enabled controlled release of rutin within acceptable limits (1.2315 µg/mm^2^ at 240 min), supporting sustained bioactive functionality without compromising structural integrity. In addition to these functional advantages, the films exhibited excellent biodegradability, strong antibacterial activity against *S. aureus* and *E. coli*, and antifungal activity against *C. albicans*. Amongst all formulations, PRC-2 demonstrated superior overall performance, notably extending the shelf life of garlic up to 23 days, highlighting its potential in active food packaging applications. Furthermore, rutin was successfully incorporated into the film matrix, and its antioxidant activity-offering a promising enhancement to the film’s functional properties-remains a key area for future investigation.

However, the study revealed certain challenges in optimizing the concentrations of CuO and rutin, it did not assess the potential migration of CuO nanoparticles. Moreover, the biodegradability tests were limited to laboratory conditions, which may not fully reflect real environmental scenarios. Overall, this study lays a strong foundation for the development of sustainable, multifunctional packaging materials and offers a promising step toward greener and safer alternatives in the food packaging industry.

## Supplementary Information

Below is the link to the electronic supplementary material.

Supplementary Material 1

## Data Availability

The authors declare that the data supporting the findings of this study are available within the paper and its Supplementary information files.

## Abbreviations

PVA: Polyvinyl alcohol
CuO: Copper oxide
NPs: Nanoparticles
PRC: Polyvinyl alcohol/Rutin/Copper oxide
Cu: Copper
Cu_2_O: Cuprous oxide
CuO: Cupric oxide
PR: Polyvinyl alcohol/rutin
MAC: Moisture adsorption capacity
WS: Water solubility
SBT: Soil burial test
CFU: Colony forming unit
WVP: Water vapor permeability
