# Supplementary material for: Antimicrobial and biodegradable PVA/rutin/CuO nanocomposite film for garlic preservation
Source: Sci Rep. 2025 Sep 29;15:33603. doi: 10.1038/s41598-025-18888-w (PMC12479998; doi:10.1038/s41598-025-18888-w)
Supplement: Supplementary file 1 — Supplementary Material 1 [file 41598_2025_18888_MOESM1_ESM.docx]

**Supplementary file**


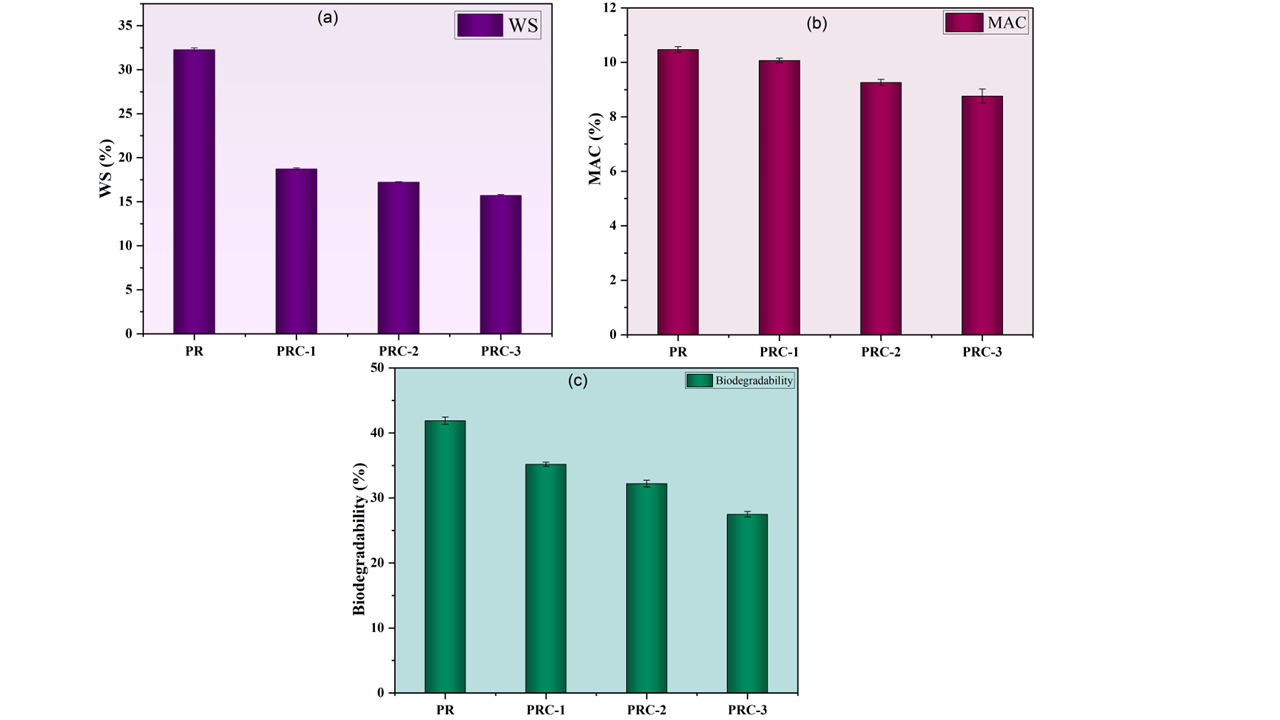


**Fig. S1.** (a) Water solubility (b) moisture adsorption capacity (c) biodegradability of the nanocomposite films

**Table S1**

Diameter of zone of inhibition of PR and PRC films

| **Sample** | **Zone of inhibition (mm)** | | |
| --- | --- | --- | --- |
|  | ***S. aureus*** | ***E. coli*** | ***C. albicans*** |
| PR | - | - | - |
| PRC-1 | 22.66±0.44 | 22.33±0.44 | 22.33±0.44 |
| PRC-2 | 24.66±0.44 | 26.66±0.44 | 24.66±0.44 |
| PRC-3 | 38.00±0.66 | 29.66±0.44 | 26.66±0.44 |
| Fluconazole (10 mcg) | - | - | 27.33±0.44 |
| SA antibacterial std (30 mcg) | 42.33±0.44 | - | - |
| EC antibacterial std (30 mcg) | - | 28.33±0.44 | - |

**Table S2**

CFU exhibited by standard, PR, and PRC films

| **Organism** | **Control** | **PR** | **PRC-1** | **PRC-2** | **PRC-3** | **Standard** |
| --- | --- | --- | --- | --- | --- | --- |
| *S. aureus* | TNTC | TNTC | 10 | 0 | 0 | 6 |
| *E. coli* | TNTC | TNTC | 152 | 0 | 0 | 0 |
| *C. albicans* | TNTC | TNTC | 0 | 0 | 0 | 79 |

TNTC: Too many numbers to count


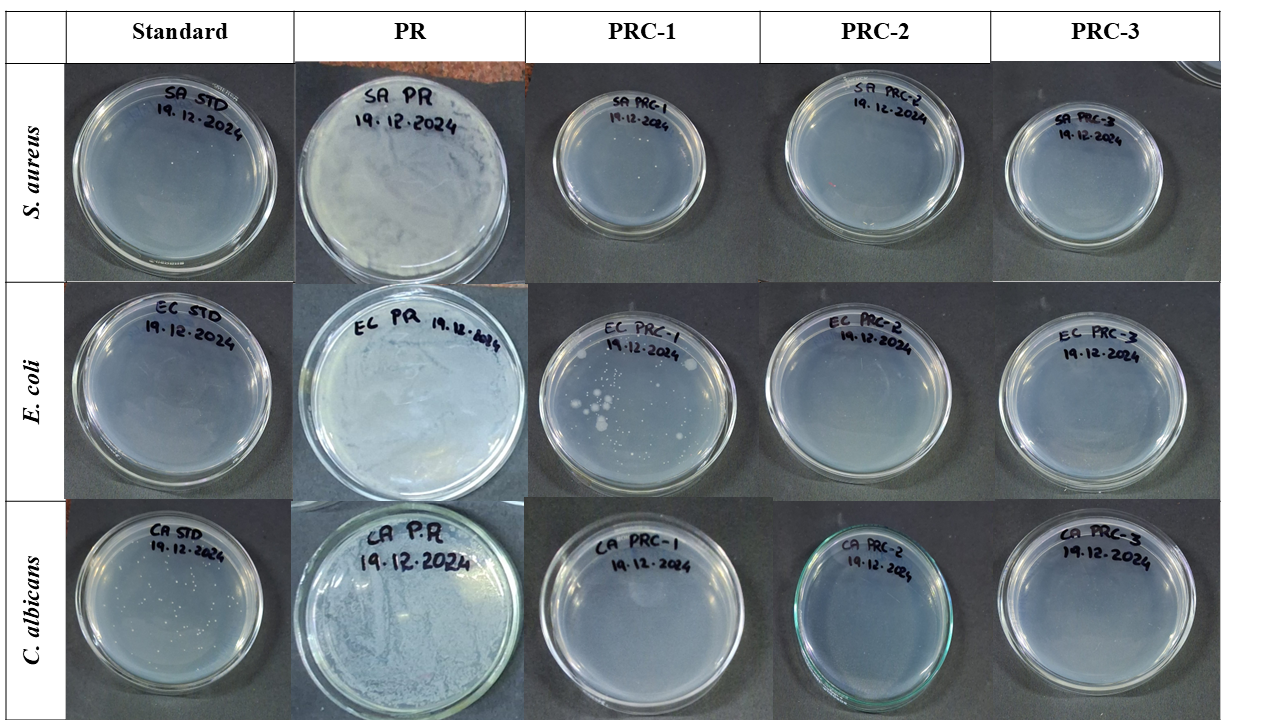


**Fig. S2.** CFU exhibited by different microorganisms
